# Progress and Problems in Bone and Mineral Disorders

**DOI:** 10.17925/EE.2017.13.01.19

**Published:** 2017-04-03

**Authors:** Neil Gittoes

**Affiliations:** Centre for Endocrinology, Diabetes and Metabolism, Queen Elizabeth Hospital, University Hospitals Birmingham and University of Birmingham, UK

**Keywords:** Osteoporosis, abaloparatide, rososozumab, hypophosphatasia, X-linked hypophosphatemia, XLH

## Abstract

A number of new drugs are moving through the osteoporosis therapy pipeline. Some show great promise for patients while one has fallen by the wayside at the last hurdle. New, effective therapies are warmly welcomed but there are still uncertainties around management of osteoporosis with currently available drugs that are contributing to what is commonly being referred to as the ‘treatment gap’; a differential between those patients who would benefit from treatment versus those who actually are receiving it. Furthermore, in parallel to the common public health disease of osteoporosis, there have been tangible developments in therapies available for some rare bone and calcium diseases.

It is highly appealing to manage osteoporosis and reduce risk of fracture by using drugs that can restore bone microarchitecture and bone strength; such drugs are bone anabolic agents. Teriparatide is the only currently licensed anabolic bone drug and has been used clinically for around 15 years,^1^ and has an important established, albeit narrow, position in the osteoporosis therapeutic armamentarium.

Abaloparatide is a 34 amino acid peptide that selectively activates the parathyroid hormone type I receptor. A daily subcutaneous injection of abaloparatide over 18 months showed a reduction in new vertebral fractures of 86% compared with placebo.^2^ Risk of non-vertebral fractures was reduced by 43%. Compared with teriparatide, bone density changes and anti-fracture effect was similar although there was a lower incidence of hypercalcaemia with abaloparatide.

Romosozumab is a humanised monoclonal antibody to sclerostin, an osteocyte-derived factor that blocks the canonical Wnt signalling pathway in bone. Romosozumab blocks sclerostin, which in turn enhances Wnt signalling, resulting in an increase in bone formation and bone mineral density. Data reveal substantial gains in bone density during romosozumab treatment and after 12 months of treatment there was a 73% reduction in new vertebral fractures compared to placebo.^3^ Clinical vertebral fractures were reduced by 36%. There was a non-significant reduction in non-vertebral fractures after 12 months of treatment. Adverse event profile was good. When compared with teriparatide, romosozumab caused greater gains in bone mineral content at the spine and the hip. Studies using sequential romosozumab with denosumab over a 2-year study period have revealed optimistic results but the exact future position of romosozumab in osteoporosis therapy remains to be determined.

In addition to the development of the two bone anabolic agents above, 2016 saw the demise of another promising anti-fracture agent called odanacatib^4^ that inhibits osteoclast function (but not viability) via inhibition of cathepsin K. Fracture prevention data were encouraging although due to a negative adverse event profile, largely around cardiovascular risk, odanacatib was pulled from further development.

While novel therapies continue to add to the therapeutic possibilities in the management of osteoporosis, it is of concern that there appears to be an ever widening treatment gap between those individuals who would benefit from therapy versus those who actually receive such therapies. This creates concern by creating a growing population of individuals at risk of fracture who are not receiving optimal care. The treatment gap has been highlighted internationally.^5^

One of the main factors in creating the treatment gap, is professional concern by physicians, as well as patient concerns relating to side effects of drugs and lack of clarity regarding long-term interventions and management of osteoporosis. Atypical femoral fractures (AFFs) and osteonecrosis of the jaw have played a centre-stage role in the lay media and professional publications that are probably responsible for over-exaggerated causes for concern. It is likely that improved education and contextualisation of information will be required to ensure that the treatment gap does not widen, but in fact, constricts. There is an onus upon us all to risk stratify with respect to potential long-term complications of antiresorptive therapy, including extended femur scans during dual-energy X-ray absorptiometry (DXA) scanning in patients being monitored with antiresorptive therapy, better identification of high-risk patients based on geometrical parameters on DXA scanning, as well as better education of patients and professionals related to prodromal symptoms, and also better research understanding of AFFs and risk factors for development.

We have developed sophisticated tools to risk stratify and identify individuals who will benefit from treatments and services, such as fracture liaison services that are in place across the globe to identify those most at risk. However, if we cannot manage the treatment gap and perceptions of risk-versus-benefit, many of these developments will not be delivered in full to the benefit of patients.

Looking at the UK Clinical Research Practice Datalink (CPRD),^6^ there was an increased rate of bisphosphonate prescriptions between 1990 and 2006 and then a plateau; however, in the last 3 years, there has been a 12% decrease in new prescriptions.^7^ These marked secular changes over the last two decades are probably revealing a change in understanding and societal views. Although, it is important to recognise that treatments, in addition to oral bisphosphonates, have become available and this is likely in small part to explain some of the more recent reductions in oral bisphosphonate prescribing.

As well as the population impact of osteoporosis in an ageing society, rare diseases have also made developments in terms of specific therapies. Hypophosphatasia is a very rare condition that has variable expression. In childhood this can be a very severe, life-threatening condition and the introduction of asfotase alfa as a recombinant mineral-targeted form of alkaline phosphatase, has revolutionised the severe end of the spectrum of disease.^8^ Its potential value on a larger scale in adults with less apparent symptomatology remains to be determined.

Finally, understanding of the aetiopathogenesis of hypophosphataemic rickets involving fibroblast growth factor (FGF) 23 signalling has allowed the evolution of anti-FGF receptor monoclonal antibodies that have been shown to improve phosphate regulation and improvement in both paediatric and adult X-linked hypophosphatemia (XLH).^9^

The field of bone and mineral metabolism is exciting and rapidly progressive. Common public health diseases continue to challenge in terms of practical management of patients, but pipeline development of new drugs continues to allow refinement and personalisation of management. In parallel, a number of the fascinating rare diseases that have been managed on an ad hoc basis to date are now starting to be managed in a targeted, disease-specific manner allowing much improved outcomes, particularly in severe disease expressed in early childhood.
